# Birt–Hogg–Dubé Syndrome: A Rare Genodermatosis Presenting as Skin Papillomas

**DOI:** 10.1093/asjof/ojad064

**Published:** 2023-07-12

**Authors:** Elina Theodorakopoulou, Alec D McCarthy, Zannis Almpanis, Shino Bay Aguilera

## Abstract

The authors present a rare case of Birt–Hogg–Dubé (BHD) syndrome that presented primarily as an aesthetic case. Previous providers failed to accurately diagnose BHD, despite the patient’s history of pneumothoraces. This female patient complained of numerous recurrent, small skin-colored growths on the face and neck and patchy hypopigmentation from the multiple treatments she had to undergo for her “bumpy skin.” She also suffered 4 spontaneous pneumothoraces. Following histopathologic and genetic testing, the patient was diagnosed with BHD. Computed tomography and ultrasound scans revealed multiple cysts in both lungs and an angiomyolipoma in both kidneys. This patient had undergone a variety of treatments to aesthetically remove and heal her skin bumps from several healthcare providers, all of whom had misdiagnosed her condition.

**Level of Evidence: 5:**

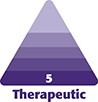

Birt–Hogg–Dubé (BHD) syndrome is a rare genodermatosis, mainly characterized by mutations of the folliculin (FLCN) gene, which is located on Chromosome 17.^1,2^ After over 40 years since its diagnostic description, the prevalence of BHD remains unknown, although it is postulated that BHD affects about 2 cases per million without prevalence toward either sex.^3^ It is usually transmitted as an autosomal-dominant variant and phenotypically presents as small benign hamartomas of the hair follicle (fibrofolliculomas), trichodiscomas, perifollicular fibromas, and acrochordons that most frequently appear on the face, neck, and upper trunk.^4,5^ Cutaneous lesions usually arise in the second through fourth decade of life, and the quantity and size of the lesions increase with age.^6,7^ The pathology may also present as spontaneous pneumothorax and is linked to an increased risk for benign and malignant kidney tumors.^8,9^ Recent evidence shows that there is a small percentage of BHD patients with negative FLCN genotype, but the same clinical phenotype, which may indicate that the condition is yet to be genetically stratified.^2^ We present a pathologically and genetically confirmed BHD case to create awareness of the inherited conditions that can present as multiple skin growths on the face and neck and can be misdiagnosed and mistreated as skin papillomas.

## CASE PRESENTATION

**Figure 1.**
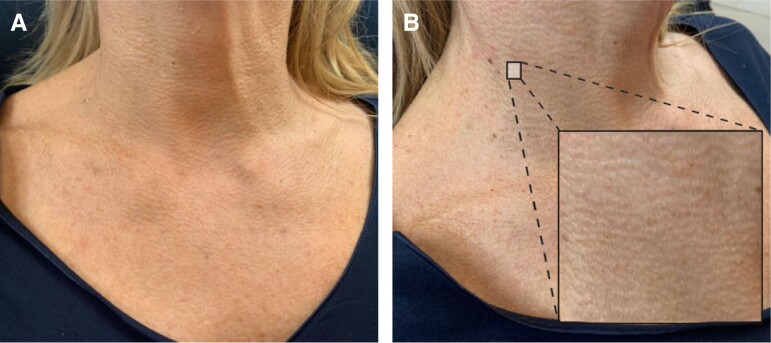
A 53-year-old female patient presenting with “bumpy neck skin.” (A) Front and (B) orthogonal view with magnified inset showing raised, hypopigmented bumps characteristic of Birt–Hogg–Dubé syndrome.

**Figure 2.**
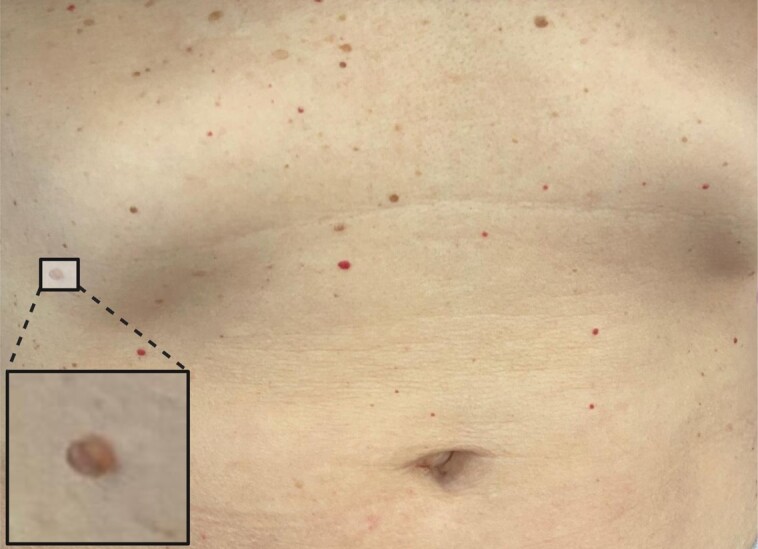
Photograph of the abdomen of the 53-year-old female patient shown in Figure 1, revealing many skin growths of different sizes and pigmentations.

A 53-year-old Caucasian female patient presented to our clinic complaining of “bumpy” skin on her face and neck, which was previously treated as common warts or skin papillomas. She noted that her skin problems started at the age of 25 and that she had attempted different treatments such as laser therapy, dermal peels, cryotherapy, and topicals. There was no clinical or laboratory indication for autoimmunity or immune deficiency. Her face was covered with patchy hypopigmented areas, perhaps associated with the various removal treatments for skin growths, including skin tags, facial warts, and skin papillomas. The skin of the face, neck (Figure 1A, B), and abdomen (Figure 2) was covered with numerous skin-colored 2 to 8 mm-diameter papules that were occasionally itchy. The presentation in this case was consistent with fibrofolliculomas reported by Tong et al^6^ In addition, several acrochordons were observed on the neck and upper chest of this patient (Figures 1B, 2). This “lumpy bumpy” appearance, especially of the neck, has always been embarrassing for the patient.

A careful medical history revealed 4 spontaneous pneumothoraces at a young age and a pleurectomy at the age of 30. She had several colon polyps removed in the past as well. Additionally, this patient revealed that she suffered from Fuchs’ Endothelial Dystrophy, which affected both eyes. She had a benign cyst surgically removed from her scalp at the age of 23 and a digital mucous cyst removed from her left hand recently. She also reports being diagnosed with polycystic ovary syndrome, for which she attributes her 7 years of fertility issues. The patient also stated that there was no known family history for BHD.

**Figure 3.**
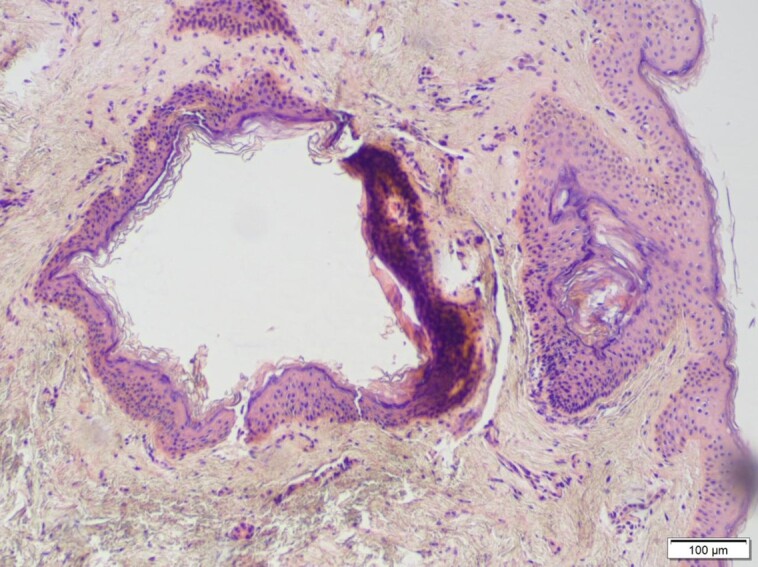
Hematoxylin and eosin stains from the biopsied tissue reveal fibrofolliculomas characterized by the presence of a dilated central follicle, loose connective tissue, and a central cyst lined by keratinizing squamous epithelium.

A small, 3 mm, flesh-colored, dome-shaped papule was surgically excised for the neck and was sent to pathology. The diagnosis was consistent with fibrofolliculoma, which appeared as a cystically dilated central follicle surrounded by loose connective tissue. In this case, the central cystic space is filled with keratinous debris and lined by keratinizing squamous epithelium that resembles normal epidermis (Figure 3). The patient underwent a full body computed tomography scan, which revealed benign cysts in both lungs and the right kidney. The clinical diagnosis of BHD was made and the patient was asked to undergo genetic testing for mutations in the 17p 11.2 FLCN gene (specifically, a duplication at cytogenetic location 17p11.2), for which she was positive, thereby confirming the diagnosis of BHD (Table). She was also referred to a lung and kidney specialist, as well as for genetic counseling.

**Table.** Genetic Testing Results Reveal Mutation of the FLCN Gene

Gene	Nucleotide change	Protein change	Heredity	Classification
FLCN	NM_144997.7_c.1285dup	p.His429ProfsTer 27	Autosomal dominant	Pathogenic

FLCN, folliculin.

## TREATMENT PROTOCOL AND RATIONALE

For her aesthetic problems, electrosurgery was recommended for the removal of the fibrofolliculomas and papillomas on the neck and abdomen, whereas for the skin laxity and pigmentation irregularities, microneedling with the application of topical exosomes was deployed. First, the mechanical perturbation of the skin from microneedling generally results in de novo elastin formation and overall skin quality improvements.^10^ Second, it creates transdermal channels to deliver exosomes.^11,12^ Exosomes have been observed, when topically applied, to improve skin quality and treat dyspigmentation.^13^ Using this combination, our aim was to treat uneven skin texture resulting from aging and past treatments of folliculofibromas. Additionally, the combination therapy was used to favorably remodel the superficial portion of the skin and overcome the presenting disease phenotype. According to previous studies, FLCN mutations in BHD modulate the mechanistic target of rapamycin complex 1 (mTORC1) and AMP-activated protein kinase (AMPK) pathways, which at the level of the skin, are important for the cellular homeostasis of fibroblasts (cell growth and proliferation).^2,14,15^ Interestingly, based on previous studies, the secretion of the endogenous exosomes is negatively impacted from alterations in the mTORC1 pathway.^16^ On another note, mesenchymal and adipose tissue–derived exosomes are able to improve wound-healing rates through activating the intracellular phosphatidylinositol 3-kinase/protein kinase B (Akt)/mTOR pathway and improve the architecture of the dermis by stimulating the production of healthy collagen and elastin.^16-18^ Moreover, an important micronutrient, called niacinamide, has been linked to improve cellular proliferation by influencing the Akt/mTOR pathways.^19^ Taken together, we believe that supplementing the skin of BHD patients with exosomes will lead to improve collagen synthesis and serve to normalize some of the skin lesions caused by the disruption on mTORC1 and AMPK.

**Figure 4.**
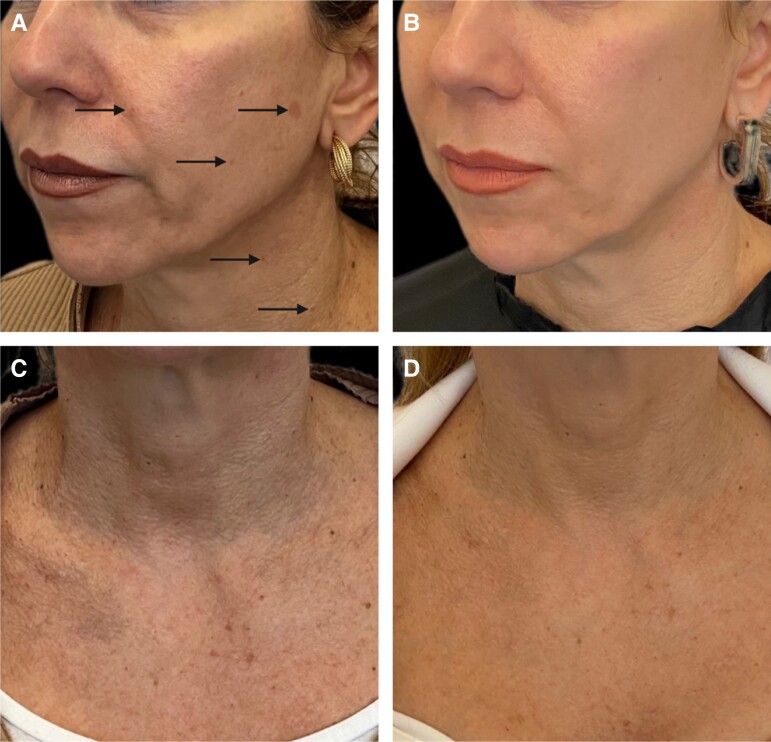
Clinical images of the 53-year-old female patient shown in Figures 1 and 2 (A, C) before and (B, D) after treatment of the face and neck, respectively.

The combination treatment protocol included 3 treatments spaced approximately 1 month apart and consisted of microneedling and topical application of exosomes as follows. First, at a depth of 0.5 to 1.0 mm, 10 passes were made with the microneedle (Dermapen 4th generation; Derma Pen, LLC; Terrey Hills, Australia) in horizontal, vertical, and oblique directions of each treatment area (face, neck, chest). During, immediately after, and 6 min post microneedling, topical exosomes (Exocode Radiance; ExoCoBio, Seoul, South Korea) were applied and gently massaged into the skin. The patient tolerated the treatment well and was satisfied with the results. Clear improvements in the degree of dyspigmentation and prevalence of papilomas can be observed (Figure 4; black arrows indicate areas of dyspigmentation with impressive resolution). The results in the face were observed faster than in the neck and décolleté, perhaps because of the lack of pilosebaceous units that promote skin repair.^10^ The patient was advised to continue the treatments for another 3 sessions, especially in the areas of neck and décolleté, after the summer period, when, from our own clinical experience, more of the BHD skin lesions arise. Following diagnosis and treatment, written and verbal informed consent and all releases for publication were obtained from the patient in accordance with regional laws in Greece.

## DISCUSSION

BHD is a rare inherited condition that often takes years before being accurately diagnosed. Because of inaccurate diagnoses, patients potentially suffer from iatrogenic complications until they are properly diagnosed, monitored, and appropriately treated. Mutations in the FLCN gene, which provide instructions for making the protein folliculin, are present in the brain, heart, placenta, testis, skin, lungs, and kidneys, and may be important for foreign particle endocytosis, which can result in a variety of benign tumors.^20^ It appears that these patients most commonly see a dermatologist for recurrent skin lesions on the face and neck, usually around the age of 20 to 30. These rare cases highlight the need to always take an extensive medical history of our patients, although they appear in our clinics for “minor problems” such as facial skin growths. However, identifying and diagnosing BHD and monitoring these patients may be crucial in sustaining their longevity. For example, the prevalence rates of pneumothorax, pulmonary cysts, renal cell carcinoma, and skin lesions are 50.9%, 91.9%, 22.5%, and 47.9%, respectively.^20,21^ These patients may also suffer from colon polyps and cancer, freckled chorioretinopathy, parotid tumors, and thyroid nodules and cancer.^22-24^ Dermoscopy and skin biopsy can always help differentiate and diagnose this rare but interesting genodermatosis. This publication is the first to report BHD associated with Fuchs disease, while confirming the association of BHD with ovarian cysts.^25,26^

## CONCLUSIONS

This case report emphasizes the need to rigorously review patient’s medical history. In this case, our patient presented with aesthetic concerns that had gone misdiagnosed and mistreated for several years. Both misdiagnoses and treatments pose a psychosocial as well as financial and temporal burden on patients. Dermatologists observing characteristic “goose skin neck” in patients between the ages of 20 and 40 and recurrent flesh-colored skin lesions on the face, neck, and upper trunk should inquire about spontaneous pneumothorax and pulmonary cysts, as they are the most commonly associated pathologies. If a provider suspects BHD, it is encouraged to perform a skin biopsy and order an appropriate genetic test.
